# Data Behind Vaccine Hesitancy and Latest Updates on Vaccines in the Pipeline—2019 Annual Conference on Vaccinology Research, April 3–5, 2019

**DOI:** 10.3201/eid2601.191246

**Published:** 2020-01

**Authors:** William Schaffner, H. Keipp B. Talbot, Marla Dalton

**Affiliations:** Vanderbilt University School of Medicine, Nashville, Tennessee, USA (W. Schaffner, H.K.B. Talbot);; National Foundation for Infectious Diseases, Bethesda, Maryland, USA (M. Dalton)

**Keywords:** Vaccine, vaccination, conference, vaccinology, National Foundation for Infectious Diseases

The field of vaccinology continues to expand and innovate in basic science discovery, product development and implementation, and evaluation of effectiveness. Innate and induced immune regulatory pathways are unraveled, new adjuvants and antigen constructs proven effective, and recently licensed products achieve high coverage, yielding noticeable decreases in disease incidence. These achievements are moving the field forward, with the expectation that many current, challenging diseases—including chronic, noninfectious, and neoplastic—might become vaccine-preventable or vaccine-treatable.

Hosted by the National Foundation for Infectious Diseases, the 2019 Annual Conference on Vaccinology Research, held in Baltimore, Maryland, USA, during April 3–5, 2019 (https://acvr.nfid.org/wp-content/uploads/2019/10/2019-ACVR-Detailed-Agenda.pdf), brought together ≈250 researchers from around the globe to discuss recent scientific advances contributing to the progress of vaccine development, evaluation, production, and implementation. The Annual Conference on Vaccinology Research provided high-quality, current reports of scientific progress and best practices through invited presentations, submitted oral presentations, and poster presentations.

Researchers across the breadth of vaccinology shared the latest updates about vaccines in the pipeline to prevent Zika, dengue, and influenza; progress to combat antimicrobial resistance; and evidence-based communications strategies to address vaccine hesitancy. By drawing on an international audience of scientists and researchers, healthcare professionals and trainees, vaccine manufacturers, and public health officials, the conference encouraged the exchange of ideas across a broad range of disciplines. Conference objectives were to discuss recent scientific advances contributing to the progress of vaccine development; identify research opportunities and scientific challenges as well as new approaches and methods associated with vaccine development, evaluation, production, and distribution; assess ethical considerations in vaccine development and clinical trials; describe effective techniques for vaccine communication; and discuss the role of vaccines in preventing existing and emerging infectious diseases and antimicrobial resistance.

Centers for Disease Control and Prevention Director Robert R. Redfield, MD, opened the conference noting, “Last season, influenza vaccination coverage decreased by more than 6 percentage points in adults, with only around 37% getting vaccinated.” He discussed the serious threat of vaccine hesitancy, which was also a recurring theme in several other presentations.

Kayvon Modjarrad, MD, PhD, of the Walter Reed Army Institute of Research then led presentations on “Rapidly Responding to Pathogen X” through implementing a forward-thinking strategy for vaccine design and development. Representatives from the Coalition for Epidemic Preparedness Initiatives, Wellcome Trust, and National Institutes of Health joined him to discuss key components of systems to successfully develop rapid countermeasures to respond to disease spillover events, opportunities to use existing and new vaccines to combat antimicrobial resistance, and ways to accelerate development by supporting flexible vaccine technology platforms.

Nicola P. Klein, MD, PhD, from the Kaiser Permanente Vaccine Study Center, moderated and presented on vaccine adverse events. She described approaches to understanding underlying mechanisms of adverse events after vaccination, ranging from investigating the individual patient to diseases and genetics in whole populations. Dr. Klein’s work discusses how genetic, immunologic, and clinical factors may predispose persons to adverse events after vaccination with measles-containing vaccines. This research may help lay the groundwork for new approaches in identifying persons who may be at risk for vaccine adverse events and improve our understanding of the interaction between vaccine safety and immune responses.

Christopher L. Karp, MD, of the Bill & Melinda Gates Foundation convened a session on advances in immunogen design, display, and formulation. Presentations reviewed the design of self-assembling protein nanoparticle vaccine candidates to elicit localized immunity to enteric pathogens, examples of germline-targeting priming immunogens to elicit broadly neutralizing antibodies against HIV, and molecular and cellular characterization of anti–*Plasmodium falciparum* circumsporozoite protein B cell memory responses to guide a next-generation malaria vaccine.

One of the highlights of the conference included a panel discussion recognizing the effect of women in vaccinology. National Foundation for Infectious Diseases Secretary H. Keipp B. Talbot, MD, MPH, moderated the discussion; panelists were Kathryn M. Edwards, MD; Julie L. Gerberding, MD, MPH; Anne Schuchat, MD; and Anita Zaidi, MBBS, SM. These 4 physician/scientists highlighted the opportunities and challenges for women in the field. After the panel discussion, they answered questions from conference attendees. This was the inaugural “women in vaccinology” event and, based on its success, will be the first of many such sessions at future conferences.

The 2020 Annual Conference on Vaccinology Research will be held in Washington, DC, March 23–25, 2020. Presentations will focus on behavioral science and vaccine hesitancy; epidemiology and burden of vaccine-preventable diseases; new vaccine delivery mechanisms; precision/personalized vaccinology; vaccine policy, programs, and practice; vaccine research, development, and production; vaccine safety and monitoring; vaccines against emerging and reemerging infectious diseases; and vaccines to combat antimicrobial resistance. Additional program and registration information is available at https://acvr.nfid.org.

